# Healthy Options: study protocol and baseline characteristics for a cluster randomized controlled trial of group psychotherapy for perinatal women living with HIV and depression in Tanzania

**DOI:** 10.1186/s12889-019-7907-6

**Published:** 2020-01-20

**Authors:** Mary C. Smith Fawzi, Hellen Siril, Elysia Larson, Zenaice Aloyce, Ricardo Araya, Anna Kaale, Janeth Kamala, Muhummed Nadeem Kasmani, Amina Komba, Anna Minja, Angelina Mwimba, Fileuka Ngakongwa, Magreat Somba, Christopher R. Sudfeld, Sylvia F. Kaaya

**Affiliations:** 1 Department of Global Health and Social Medicine, Harvard Medical School, 641 Huntington Ave., Boston, MA 02115 USA; 2 Management and Development for Health, Plot #802 Mwai Kibaki Road, Mikocheni, Dar es Salaam, Tanzania; 3 Department of Global Health and Population, Harvard T.H. Chan School of Public Health, 655 Huntington Ave, Boston, MA 02115 USA; 4 Africa Academy for Public Health, Plot #802 Mwai Kibaki Road, Mikocheni, Dar es Salaam, Tanzania; 5 Kings College London, De Crespigny Park, London, SE5 8AF UK; 6 Department of Psychiatry and Mental Health, Muhimbili University of Health and Allied Sciences, United Nations Road, Dar es Salaam, Tanzania

**Keywords:** HIV, Depression, PMTCT, Women, Intervention, Treatment

## Abstract

**Background:**

Perinatal women accessing prevention of mother-to-child transmission of HIV (PMTCT) services are at an increased risk of depression; however, in Tanzania there is limited access to services provided by mental health professionals. This paper presents a protocol and baseline characteristics for a study evaluating a psychosocial support group intervention facilitated by lay community-based health workers (CBHWs) for perinatal women living with HIV and depression in Dar es Salaam.

**Methods:**

A cluster randomized controlled trial (RCT) is conducted comparing: 1) a psychosocial support group intervention; and 2) improved standard of mental health care. The study is implemented in reproductive and child health (RCH) centers providing PMTCT services. Baseline characteristics are presented by comparing sociodemographic characteristics and primary as well as secondary outcomes for the trial for intervention and control groups. The trial is registered under clinicaltrials.gov (NCT02039973).

**Results:**

Among 742 women enrolled, baseline characteristics were comparable for intervention and control groups, although more women in the control group had completed secondary school (25.2% versus 18.2%). Overall, findings suggest that the population is highly vulnerable with over 45% demonstrating food insecurity and 17% reporting intimate partner violence in the past 6 months.

**Conclusions:**

Baseline characteristics for the cluster RCT were comparable for intervention and control groups. The trial will examine the effectiveness of a psychosocial support group intervention for the treatment of depression among women living with HIV accessing PMTCT services. A reduction in the burden of depression in this vulnerable population has implications in the short-term for improved HIV-related outcomes and for potential long-term effects on child growth and development.

**Trial registration:**

The trial is registered under clinicaltrials.gov (NCT02039973). Retrospectively registered on January 20, 2014.

## Background

Major depression has progressively accounted for a greater burden of disability worldwide, as reflected in estimated increases in disability-adjusted life years (DALYs) [1]. Recent studies have documented the substantial burden of depression in resource-limited settings, which can result in not only significant disability, but also premature mortality [2]. Depression is often a co-morbid condition with other chronic illnesses, such as cancer, diabetes, and HIV [3–5]. In a meta-analysis of ten studies, people living with HIV experienced a two-fold increase in risk of major depressive disorder (MDD) compared to those who were HIV-negative [6]. Comorbidity of HIV and depression has direct implications for the risk of mortality and poor prognosis [7, 8]. Therefore, it is critical to identify and treat depression among people living with HIV (PLH) to improve HIV-related outcomes as well as overall health and well-being.

Women accessing prevention of mother-to-child transmission of HIV (PMTCT) services face the same set of challenges as other PLH; however, a number of additional factors can place these women at even higher risk of depression. In resource-limited settings women often have lower access to formal education and fewer opportunities for employment as compared with men, which increases vulnerability for depression and intimate partner violence [9–11]. For example, in The Gambia women living with HIV without an independent income were at an increased risk of elevated depressive symptoms as compared to women with an independent income [12]. Women that are economically dependent on their partners are also at significant risk of intimate partner violence [13], which is strongly associated with major depression [14, 15]. In addition, women have a greater risk of depression during the perinatal period, which has implications for their own health outcomes as well as the health and well-being of their infants [16–19]. In addition to intimate partner violence, other relevant risk factors associated with depression among women living with HIV include non-adherence to ART and HIV-related stigma [20, 21], both of which also contribute to poor clinical outcomes for HIV. In contrast, relevant protective factors related to depression as well as HIV-related clinical outcomes include social support, hope, and self-efficacy [22–24].

In Tanzania, the prevalence of symptoms consistent with MDD among women accessing PMTCT services in Dar es Salaam has been estimated as greater than 40% [8]. However, antenatal care services in Tanzania are typically offered by nurse-midwives, who have limited skills for assessment and intervention among women with major depression. This is compounded by the fact that there is a significant shortage of health professionals in Tanzania, particularly those with mental health care expertise which is estimated at 0.3 per 100,000 population (including psychiatric nurses, psychiatrists, social workers, psychologists, and other mental health workers with formal training) [25].

To address the gap between the need for treatment of depression and available services, a number of resource-limited settings have utilized a task-sharing approach where lay or non-specialist providers are trained to deliver psychosocial interventions for the treatment of depression. For example, in Pakistan, Lady Health Workers were trained to deliver a cognitive behavioral intervention with perinatal women and demonstrated a significantly lower prevalence of depression in the intervention group as compared with controls [26]. In Zimbabwe, the “Friendship Bench” program worked with lay health workers and trained them to provide six sessions of problem-solving therapy (PST) as well as screen and monitor symptoms of common mental disorders [27]. Additionally, women receiving primary health care in KwaZulu-Natal, South Africa, were enrolled in a feasibility study to examine initial outcomes related to provision of Interpersonal Therapy (IPT) for depression facilitated by community health workers (CHWs); a reduction in depressive symptoms was observed upon completion of the intervention and was sustained after 24 weeks of follow-up [28]. In a more recent study among individuals receiving primary health care in South Africa, those receiving a task-sharing counselling intervention delivered by nurses experienced a lower level of depressive symptoms and improvement in WHO Disability Assessment Scale (WHODAS) scores [29]. In a randomized controlled trial in Pakistan women in the intervention group received an adapted version of the WHO Program Management Plus program with Lady Health Workers facilitating psychosocial support group sessions. Improvements were observed across a number of outcomes, including symptoms of anxiety and depression, WHODAS scores, as well as general psychological distress [30]. Although the evidence has been expanding, this task-sharing approach to addressing depression has not been evaluated in Tanzania or among women accessing PMTCT services more broadly.

In order to address the burden of depression and limitation in access to services among perinatal women living with HIV we are implementing a study to compare treatment strategies for depression to advance evidence-based care in this vulnerable population. Utilizing a cluster randomized controlled trial study design we are comparing a task-sharing approach (i.e. problem-solving and cognitive behavioral therapy components delivered to groups facilitated by trained lay community-based health care workers) to improved standard of care for the treatment of depression among women living with HIV accessing PMTCT services in Dar es Salaam, Tanzania. In this paper we present the study protocol and report the baseline characteristics of the trial population.

## Methods

### Study setting

This study is being conducted within three urban districts in Dar es Salaam, Tanzania: Ilala, Kinondoni, and Temeke. According to the 2015 Demographic and Health Survey (DHS), 96% of women aged 15–49 in Dar es Salaam had attended school, with 44% completing secondary school. Sixty-six percent of women were employed, with the most common employment being unskilled manual labor and domestic service [31]. The prevalence of HIV among pregnant women in Dar es Salaam has been estimated as 6.6% [32]. Study sites are government-run reproductive and child health (RCH) clinics in Dar es Salaam that provide a broad range of prevention of mother-to-child transmission of HIV (PMTCT)-related services including antiretroviral therapy (ART), counseling, as well as provision of nevirapine and HIV testing for infants at 6 weeks of age.

### Trial design and randomization

The study is a cluster randomized controlled trial with clusters randomized to intervention or control arms with a 1:1 allocation ratio. Clusters were pair-matched by geographic location within districts (Ilala, Kinondoni, and Temeke). Within each pair match we used a random number generator to determine which cluster would be the intervention cluster. Each cluster was defined as a primary health care facility with a RCH clinic that provided PMTCT services and its satellite facilities. Satellite facilities were those that referred patients to the primary health facility and were therefore administratively and clinically linked. Ten of the clusters had a single satellite facility and the remaining six did not have satellite facilities.

Healthcare facilities were eligible for inclusion in this study if they were government-managed RCH clinics in Dar es Salaam that were supported by a local non-governmental organization, Management and Development for Health (MDH). At the time of facility selection, MDH supported 203 of the 231 active RCH clinics in the region (88%). Facilities were eligible for inclusion in the study if they were dispensaries or health centers (i.e. hospitals, *N* = 3, were excluded), and if they saw at least 350 HIV-positive pregnant women in the year prior to the start of the study. Facility selection occurred in two rounds. In the first round of selection in March 2015 12 facilities were selected randomly, matched, and randomized. In November 2015 an additional four facilities were selected, matched, and randomized. A description of the study facilities can be found in Table 1.

**Table 1:** Description of study clusters

Cluster	Number of facilities	Intervention status	Type of facility^a^	Number of clinicians	New ANC clients^b^	HIV-infected clients at first ANC^b^	Mean baseline PHQ-9 score
Kinondoni							
Dhakiya^c^	1	Intervention	Dispensary	Medical Doctors: 0 Other Clinicians^d^: 10	219	7	11.5
Imani	2^e^	Control	Dispensary	Medical Doctors: 0 Other Clinicians: 12	127	10	11.2
Syandene	1	Intervention	Health center	Medical Doctors: 1 Other Clinicians: 55	340	15	11.6
Saida	2	Control	Health center upgraded to Hospital (2016)	Medical Doctors: 19 Other Clinicians: 176	263	25	11.5
Ilala							
Nuru	2	Intervention	Health center	Medical Doctors: 1 Other Clinicians: 63	436	20	10.6
Aadila	1	Control	Health center upgraded to Hospital (2015)	Medical Doctors: 11 Other Clinicians: 160	518	41	10.9
Firyali	1	Intervention	Dispensary	Medical Doctors: 0 Other Clinicians: 44	228	11	11.5
Kanoni	2	Control	Dispensary	Medical Doctors: 2 Other Clinicians: 28	148	6	10.2
Tumpe	2	Intervention	Health Center	Medical Doctors: 0 Other Clinicians: 43	147	20	9.8
Sekelaga	2	Control	Health Center	Medical Doctors: 0 Other Clinicians: 19	265	6	10.3
Temeke							
Amidah	1	Intervention	Was a health center upgraded to Hospital (2015)	Medical Doctors: 13 Other Clinicians: 127	520	17	11.7
Zahra	1	Control	Health center	Medical Doctors: 5 Other Clinicians: 40	482	23	12.4
Farida	2	Intervention	Dispensary	Medical Doctors: 0 Other Clinicians: 29	283	11	11.5
Tusajigwe	2	Control	Dispensary (Faith based but under PEFERF through MDH support)	Medical Doctors: 1 Other Clinicians: 13	106	4	13.0
Mansa	2	Intervention	Health center	Medical Doctors: 0 Other Clinicians: 48	139	7	10.3
Zuwena	2	Control	Dispensary	Medical Doctors: 0 Other Clinicians: 24	135	10	12.0

^a^Dispensaries are primary healthcare clinics that offer basic outpatient and maternal health services. Health centers offer both outpatient and inpatient care, including maternal and child health services. They are the first level of referral from dispensaries

^b^Monthly averages for 2014

^c^Clinic names are pseudonyms

^d^“Other clinicians” includes clinical officers, nurses, and medical attendants

^e^When there are multiple facilities within the cluster, the following columns describe the primary facility in that cluster

Initial cluster selection and randomization occurred in March 2015 with 12 facilities. There were three changes in the selection of clusters after this initial randomization. First, 2 weeks after randomization one of the control sites was upgraded from a health center to a hospital, and was therefore not eligible to be a study site. The RCH clinic was moved to a nearby health center and this new health center thus replaced the original one as the control facility for this study. Second, in November 2015, there were fewer eligible women than expected identified in the study facilities. The study team thus identified, matched, and randomized an additional four facilities from the list of original list of eligible facilities. Third, in January 2016 one of the intervention facilities also was noted to have low enrollment due to fewer than expected women eligible at that site. Therefore, a new facility was randomly selected to replace the intervention facility from the two eligible facilities identified. This resulted in 16 study sites/clusters as compared to the original 12 sites that were planned.

### Study participant selection and recruitment

Within the selected study clusters, women were eligible for participation if they were at least 18 years old, up to 30 weeks of gestation, HIV-positive and receiving ART, demonstrated symptoms consistent with MDD on the Patient Health Questionnaire-9 (PHQ-9) of a score of ‘9’ or above, planned to continue their post-partum care at the study facility, and without a current plan to harm herself, including the intent to commit suicide. A cut-off score of ‘9’ for the PHQ-9 was used based on the results from a prior validation study in Tanzania [33]. If the woman had suicidal thoughts a formal referral to the psychiatric nurse at the facility was made. Women were recruited from the study facilities from May 25, 2015 until the target sample size was reached on April 29, 2016. Research assistants conducted recruitment by approaching women attending the PMTCT clinics at these sites. All women who were pregnant and HIV-positive were approached and invited to participate after an interview to determine their eligibility. Women who had received their HIV-positive diagnosis on the day of recruitment were contacted 2 weeks later to determine eligibility. If eligible, women were invited to participate in the study.

### Intervention and control conditions

The Healthy Options intervention includes evidence-based components from problem-solving therapy (PST) and cognitive behavioral therapy (CBT) approaches [34, 35]. The PST component includes six psychosocial group therapy sessions plus one orientation session prior to delivery. Implementation strategies for the PST sessions are borrowed from *NAMWEZA* [36], a participatory and strengths-based program that was adapted for an urban Tanzanian context and demonstrated improved clinical outcomes in PLH [37]. This approach promotes awareness of one’s own strengths and assertive communication skills. The content of PST sessions includes acknowledging problems and identifying strategies for addressing them, focusing on positive thinking approaches, planning for the future and dreaming/fostering hope, as well as assertiveness and goal setting.

During the follow-up interview 6 weeks after delivery, women with elevated depressive symptoms consistent with a diagnosis of MDD (scoring ‘9’ or above on the PHQ-9) are invited to participate in eight CBT group sessions. The CBT sessions were adapted from the evidence-based “Thinking Healthy” intervention for psychosocial management of perinatal depression [38] for delivery in small groups of 8–10 women and include: discussing the link between one’s thoughts and feelings; sharing practical skills of caring for their infants and addressing daily challenges; discussing strategies for managing stress; fostering support from their social networks; and offering feedback on their CBT homework assignments with the goal of addressing depressed mood through cognitive restructuring.

Psychosocial support group sessions are facilitated in Swahili by lay community-based health care workers (CBHWs) who had prior experience with providing HIV-related peer counseling and support. A two-week in-depth training included didactic and experiential components whereby the CBHWs played the role of study participants in each of the sessions, with trainers serving as facilitators. The group sessions are approximately 2–3 h in duration and include 15–20 women, with smaller break-out groups for discussion and skills building, and are held in a variety of venues, such as health facilities, schools, and religious sites.

The control condition, referred to as “improved standard of care”, is a one-day training session offered to clinical staff including medical officers, clinical officers, nurses, medical social workers, and medical attendants at the RCH clinics where the study was recruiting participants. The training relies on evidence-based WHO mhGAP guidelines [39], focusing on: screening for depression; using an algorithm for assessment and management; attending to aspects of depression that can be addressed in a primary care setting, including exploration of current stressors, psychoeducation, reactivating social networks, offering structured physical activity, and follow-up; and identifying patients that need referral for specialized psychiatric care. This included access to counseling as well as anti-depressant treatment when necessary, as well as immediate care for individuals presenting with acute suicidal ideation. In the event of acute risk of suicide, study participants are escorted to a facility that offers services for further evaluation of suicidality by appropriately trained mental health professionals. This training session is also offered to the intervention sites and is designed to improve the capacity of all of the facilities to provide screening, treatment, and referral for women presenting with elevated levels of depressive symptoms.

### Measures

The primary outcome of interest is a level of depressive symptoms consistent with MDD on the PHQ-9 [40] measured at baseline, 6 weeks and 9 months post-partum. A prior validation study was performed in a primary health care setting in Tanzania, which demonstrated that a lower cut-off score of ‘9’ maximized the sensitivity and specificity for identifying major depression in this setting. Therefore, a score of ‘9’ or above was used to reflect an elevated level of depressive symptoms consistent with major depression in this study [33]. The PHQ-9 has demonstrated good internal consistency (Cronbach’s alpha ranging from 0.86 to 0.89) and criterion validity (sensitivity and specificity of 88%). Secondary outcomes include intimate partner violence (IPV), social support, self-efficacy, hope, HIV-related stigma, and ART adherence. IPV is defined as report of either physical and/or sexual violence and items are derived from the DHS [41]. For social support the Duke University-UNC Functional Social Support Questionnaire is used; this scale has demonstrated positive correlations with other social support scales. A listing of ten items from this questionnaire has been used previously in Tanzania [8] and includes dimensions of instrumental and emotional support [42]. The General Self-Efficacy Scale is used to assess self-efficacy. This scale has demonstrated good internal consistency (Cronbach’s alpha ranging from 0.81 to 0.91) and construct validity has been shown through positive correlations with optimism and negative correlations with depression and anxiety [43]. Hope is assessed through a scale developed locally by Siril et al. [44] with individuals living with HIV in Tanzania. For HIV-related stigma, the HIV Stigma Scale developed by Berger et al. [45] is used. This measure was validated in a diverse sample in the U.S. and demonstrated construct validity through factor analysis and internal consistency (Cronbach’s alpha of 0.96). A sub-sample of these items also demonstrated construct validity of this scale in a resource-limited setting [46]. In addition, we assess self-reported ART non-adherence as the proportion of women who had missed at least 1 day of ART in the past 4 days [47, 48]. Finally, in order to assess women’s food security, an important indication of their current economic standing, we use a composite measure. Women are considered food insecure if they report that their household had any trouble satisfying their food needs in the past 6 months and/or they currently owe money at a shop where they purchased food.

### Baseline data collection and follow-up

Trained research assistants conducted structured eligibility and baseline surveys from May 2015 through April 2016. The eligibility form consisted of items indicated in the inclusion and exclusion criteria, including age, number of weeks of gestation, information regarding whether the woman planned to receive post-natal care at the same facility for 2 years after birth, and the depression screening questionnaire (PHQ-9). Prior to the interview all women provided written informed consent. Surveys were conducted in person and lasted approximately 30 min. Blinded data collection was not feasible given the clinic-based cluster design. Responses were recorded on paper and entered into a Microsoft Access database. Data were reviewed weekly for errors and inconsistencies and corrections were made as necessary. Follow-up data are collected at 6 weeks and 9 months after delivery. The follow-up data are collected electronically on smart phones. All data are exported to Stata version 14.2 for analysis (StataCorp LP, College Station, USA) [49].

### Sample size and statistical analysis

Power calculations were conducted assuming randomization of 12 clusters, stratified by district, and pair-matched. We planned to screen 60 patients in each cluster. Given experience with other trials we assumed that 80% of patients would be enrolled from each cluster and a conservative 30% would drop out throughout the study, we anticipated having 34 patients in each cluster at the end of the study. Given this sample size (720 women screened), an anticipated depression prevalence of 73% in the control arm and 60% in the intervention arm 6 months after completion of the intervention [34], as well as a coefficient of variation of 0.05, the minimum detectable risk ratio for 80% power and 5% two-sided type I error was 0.79 [50]. With the addition of clusters after initial randomization, our power increased.

In this initial paper we present the baseline characteristics of participants in the intervention and control clusters. We include sociodemographic characteristics of participants as well as the baseline values for the primary and secondary outcomes of interest for the trial. The primary outcome will be depressive symptoms consistent with MDD. Secondary outcomes will include general self-efficacy, social support, HIV-related stigma, intimate partner violence, hope, and ART adherence. We present proportions for all categorical variables and the mean and standard deviation for all continuous variables and scales. Future analyses will determine the effect of the intervention on these primary and secondary outcomes.

For the outcomes analysis, an intent-to-treat strategy will be employed, consistent with what is typically performed for randomized controlled trials, with the primary outcome being depressive symptoms at follow-up. Secondary outcomes will include intimate partner violence, social support, self-efficacy, hope, HIV-related stigma, and ART adherence. Generalized estimating equations (GEE) will be used to compare outcomes for intervention and control groups at first and second follow-up assessments at 6 weeks and 9 months post-delivery, respectively. In order to account for the clustering of data we will utilize robust standard errors for GEE models. A sensitivity analysis will be performed to adjust for variables that have demonstrated some imbalance at baseline, such as education and marital status. Additional sensitivity analyses will be performed, including a pair-matched analysis as well as a non-parametric analysis to account for variables that are not normally distributed. Effect modification for the main outcome variable of depression will also be explored and will include variables such as baseline depression score, intimate partner violence, HIV status disclosure to partner, social support, HIV-related stigma, and hope. A parallel analytic strategy will be performed for all secondary outcomes.

## Results

Of the 895 pregnant, HIV-positive women approached 75 (8.4%) were excluded because their gestational age was beyond 30 weeks and two (0.2%) declined to participate. There were thus 818 women who participated in the PHQ-9 screening. Of those screened, 742 (90.7%) were determined to be eligible for the study based on a PHQ-9 score of 9 or above. Therefore, the overall ineligibility rate among the initial 895 women approached was 16.9% based on gestational age and PHQ-9 score. Baseline results suggest that this is a vulnerable population with approximately one-third reporting their household had any trouble satisfying their food needs in the past 6 months and 28.5% reporting that they owed money at the shop where they purchase food. This resulted in 45.4% of women being food insecure, i.e. reporting positive for at least one food insecurity item. In addition, approximately 65% of the study population had an education at the primary school level or lower. Only 12 and 17% had a flush toilet and running water in their homes, respectively.

Women enrolled in the intervention and control facilities were similar in almost all variables, with the exception of marital status and education (Table 2). A larger proportion of women in the control group had completed secondary school (25.2% versus 18.2% in the intervention group) and a lower percent were married (39.5% versus 48.1 in the intervention group).

**Table 2:** Baseline socio-demographic characteristics of the study population

	Control (*N* = 347) n (%)	Intervention (*N* = 395) n (%)
Demographic		
Age, mean (SD)	29.5 (5.3)	29.8 (5.5)
Marital status		
Married	136 (39.5%)	190 (48.1%)
Cohabitating with proposal for marriage	78 (22.7%)	67 (17.0%)
Cohabitating without proposal for marriage	30 (8.7%)	35 (8.9%)
Single	77 (22.4%)	88 (22.3%)
Divorced/separated	22 (6.4%)	13 (3.3%)
Widowed	1 (0.3%)	2 (0.5%)
Highest level of education completed		
Less than primary school	46 (13.3%)	52 (13.3%)
Primary school	212 (61.4%)	268 (68.5%)
Secondary school	87 (25.2%)	71 (18.2%)
Employment status		
Employed	41 (11.8%)	36 (9.1%)
Self-employed	155 (44.8%)	164 (41.6%)
Homemaker	147 (42.5%)	191 (48.5%)
Student	1 (0.3%)	2 (0.5%)
None	2 (0.6%)	1 (0.3%)
Household economic indicators		
Main source of energy for lighting		
Electricity	265 (76.4%)	282 (71.4%)
Solar	14 (4.0%)	13 (3.3%)
Other	68 (19.6%)	100 (25.3%)
Household floor made from cement or stone	343 (98.8%)	382 (96.7%)
Main source of drinking water		
Piped into dwelling/plot	73 (21.0%)	53 (13.5%)
Public/neighbors tap	149 (42.9%)	189 (48.0%)
Water vendor	109 (31.4%)	99 (25.2%)
Other	16 (4.6%)	53 (13.5%)
Toilet		
Flush toilet used by household/family only	50 (14.6%)	38 (9.7%)
Pit latrine used by household/family only	32 (9.3%)	63 (16.2%)
Flush toilet shared with other tenants	110 (32.1%)	101 (25.9%)
Pit Latrine shared with other tenants	139 (40.5%)	180 (46.2%)
Flush toilet shared with a neighbor	12 (3.5%)	8 (2.1%)
Had problems satisfying food needs in past 6 months	128 (37.0%)	118 (29.9%)
Owed money to shops where they purchase food	99 (28.5%)	103 (26.1%)

Scores for the primary and secondary outcomes were also fairly similar at baseline across intervention and control groups (Table 3). The findings reflected the vulnerability of the study population with 17% experiencing intimate partner violence in the past 6 months. The average social support scale score of ‘3’ suggests that this population had moderate, but less than optimal social support at baseline, since a score of ‘3’ corresponds with the response that she received support ‘less than I would like.’ In contrast, general self-efficacy was good, since women reported ‘moderately true’ on average for items reflecting self-efficacy at baseline.

**Table 3:** Primary and secondary outcomes for the randomized controlled trial at baseline (*n* = 742)

	Control *n* = 347	Intervention *n* = 395
Depression (possible range 1–27; mean, SD)^a^	11.6 (3.0)	11.3 (3.2)
Any intimate partner violence^b^	53 (17.4%)	59 (16.3%)
Social support scale (range 1–4; mean, SD)^c^	3.0 (0.7)	2.9 (0.7)
Self-efficacy scale (range 1–4; mean, SD)^c^	3.1 (0.6)	3.1 (0.8)
Hope scale (range 1–4; mean, SD)^c^	3.5 (0.4)	3.5 (0.4)
HIV-related stigma scale (range 1–5; mean, SD)^d,e^	2.2 (0.7)	2.0 (0.7)
Missed one or more days of ART in past 4 days	44 (13.3%)	58 (14.7%)

the N for individual questions may differ from the overall N due to question non-response or, where noted, due to eligibility to answer the question

^a^Lower values are better; women were screened prior to enrollment and eligible if their score on the screening survey was 9 or higher

^b^659 women (305 in control facilities and 363 in intervention facilities) reported being in an intimate partner relationship and/or chose to answer this question

^c^Higher values are better

^d^Lower values are better

^e^548 women reported disclosing their HIV status to anyone; n for this scale is 484

## Discussion

Access to treatment for depression among people living with HIV has the potential to improve prognosis and enhance survival [51, 52]. For women accessing PMTCT services, the benefits can also extend to their infants, since perinatal depression among women has demonstrated a negative impact on early child growth and cognitive development, which has short- as well as long-term implications [53]. The present cluster randomized controlled trial will compare two strategies for the treatment of symptoms consistent with MDD among women accessing PMTCT services in Dar es Salaam. Findings from the trial will inform approaches for addressing depression in this high-risk population in Tanzania and similar resource-limited settings.

At baseline, this population of women living with HIV and depression accessing PMTCT services had limited educational attainment and employment. This context of socioeconomic vulnerability may place women living with HIV who are also expecting a child at increased risk for depression. This is compounded by a significant level of food insecurity, risk of intimate partner violence, as well as the broader context of HIV-related stigma experienced by this population, factors that have also demonstrated associations with depression [54]. Prior studies have also found that PLH in other resource-limited settings are vulnerable to a similar set of economic and social conditions that may place them at risk of depression. Among PLH accessing services at an ART clinic in Ethiopia, food insecurity and not owning livestock increased the risk of elevated depressive symptoms, whereas having attended secondary school conferred a protective effect [55]. In rural Uganda, severe food insecurity was associated with elevated depressive symptoms among women living with HIV, with social support serving as a buffer in this relationship [56].

Intimate partner violence can occur within the context of HIV disclosure and can range from forcing the woman from leaving the house, intentionally disclosing her HIV status to others, as well as sexual and physical abuse, which in turn can increase the risk of depression [14, 15]. In addition, disclosure can pose a significant risk in light of the stigma that surrounds HIV. Although ART is more widely available, HIV-related stigma remains, often having a profound effect on PLH and can serve as an additional risk factor for depression [57, 58].

However, major depression is a treatable condition with psychotherapeutic approaches and/or anti-depressant medication. Lifting the burden of depression may result in women having greater capacity to engage in economic opportunities, such as investment in small businesses through collective microfinancing [59]. Similarly, women can more effectively address HIV-related stigma within the social context as well as confront issues of domestic violence in their lives if depression is addressed [60]. This in turn may not only have a positive effect on women’s health and well-being, but also for children’s cognitive and physical health outcomes [61].

There are a number of limitations of this study. Given the limited number of clusters, there is the potential of not finding associations where they may exist, particularly for secondary outcomes. In addition, the present research only recruits women accessing PMTCT services prior to 30 weeks of gestation. It is likely that women that are accessing care later, or not accessing antenatal and PMTCT services at all, may be more likely to be at risk of depression or may be suffering from more severe levels of depressive symptoms. Therefore, findings from this study cannot be generalized to those not accessing these services or accessing them at a later gestational age. Additionally, since the study was performed within the context of PMTCT services, some women recruited for the study had recently received their HIV-positive diagnosis. This can result in a higher percentage of women eligible for the study if some women experienced depressive symptoms related to the immediacy in which they received their HIV diagnosis. Although this may limit the generalizability of the study, it is not a threat to the internal validity, since a comparable recruitment strategy was used for intervention and control sites. Despite these limitations, this study is an initial step in identifying evidence-based strategies that can address depression in this context. A strength of this study is evident in the preliminary findings, whereby socioeconomic factors and other baseline characteristics demonstrated similar distributions at baseline between the intervention and control groups.

## Conclusions

In summary, treatment of depression is an essential building block in reducing the risk of poor HIV-related health outcomes and addressing the socioeconomic contexts that often contribute to the risk of depression. The potential implications are short-term, in the sense that treatment of depression can enhance a woman’s capacity to address immediate challenges, such as adhering to HIV medication, finding employment, and securing food for her family. In addition, the long-term implications are significant, since maternal depression can have an impact on cognitive development, educational outcomes, and economic attainment of their children as they transition into adulthood. Therefore, identifying evidence-based approaches for the treatment of depression among perinatal women living with HIV in resource-limited settings is critical to inform future programs and policies that aim to address the problems faced by this vulnerable population in Tanzania and similar resource-limited settings.

## Data Availability

The datasets used and/or analysed during the current study are available from the corresponding author on reasonable request.

## Abbreviations

ANC: Antenatal clinic
ART: Antiretroviral therapy
CBHW: Community-based health worker
CBT: Cognitive Behavioral Therapy
DALYs: Disability-adjusted life years
DHS: Demographic and Health Survey
IPV: Intimate partner violence
MDD: Major depressive disorder
MDH: Management and Development for Health
PHQ-9: Patient Health Questionnaire-9
PLH: People living with HIV
PMTCT: Prevention of mother-to-child transmission of HIV
PST: Problem-solving therapy
RCH: Reproductive and Child Health Clinic
RCT: Randomized controlled trial
SD: Standard deviation

## References

[1] Murray CJL, Vos T, Lozano R (2012). Disability-adjusted life years (DALYs) for 291 diseases and injuries in 21 regions, 1990–2010: a systematic analysis for the Global Burden of Disease Study 2010. Lancet.

[2] Wulsin LR, Vaillant GE, Wells VE (1999). A systematic review of the mortality of depression. Psychosom Med.

[3] Golden SH, Lazo M, Carnethon M, Bertoni AG, Schreiner PJ, Roux AVD (2008). Examining a bidirectional association between depressive symptoms and diabetes. JAMA.

[4] Smit M, Olney J, Ford NP (2018). The growing burden of noncommunicable disease among persons living with HIV in Zimbabwe. AIDS.

[5] Krebber AMH, Buffart LM, Kleijn G (2014). Prevalence of depression in cancer patients: a meta-analysis of diagnostic interviews and self-report instruments. Psychooncology.

[6] Ciesla JA, Roberts JE (2001). Meta-analysis of the relationship between HIV infection and risk for depressive disorders. Am J Psychiatry.

[7] Sudfeld CR, Kaaya S, Gunaratna NS (2017). Depression at antiretroviral therapy initiation and clinical outcomes among a cohort of Tanzanian women living with HIV. AIDS.

[8] Antelman G, Kaaya S, Wei R (2007). Depressive symptoms increase risk of HIV disease progression and mortality among women in Tanzania. JAIDS.

[9] Hoebel J, Maske UE, Zeeb H, Lampert T (2017). Social inequalities and depressive symptoms in adults: The role of objective and subjective socioeconomic status. PLoS One.

[10] Lund C, Breen A, Flisher AJ, Kakuma R, Corrigall J, Joska JA (2010). Poverty and common mental disorders in low and middle income countries: a systematic review. Soc Sci Med.

[11] Yari A, Nouri R, Rashidian H, Nadrian H (2013). Prevalence and determinants of sexual intimate partner violence against women in the city of Marivan, Iran. J Family Reprod Health.

[12] Klis S, Velding K, Gidron Y, Peterson K (2011). Posttraumatic stress and depressive symptoms among people living with HIV in the Gambia. AIDS Care.

[13] Dhungel S, Dhungel P, Dhital SR, Stock C (2017). Is economic dependence on the husband a risk factor for intimate partner violence against female factory workers in Nepal?. BMC Womens Health.

[14] Gibbs A, Dunkle K, Jewkes R (2018). Emotional and economic intimate partner violence as key drivers of depression and suicidal ideation: a cross-sectional study among young women in informal settlements in South Africa. PLoS One.

[15] Devries KM, Mak JY, Bacchus LJ (2013). Intimate partner violence and incident depressive symptoms and suicide attempts: a systematic review of longitudinal studies. PLoS Med.

[16] Stewart RC (2007). Maternal depression and infant growth – a review of recent evidence. Matern Child Nutr.

[17] Rahman A, Bunn J, Lovel H, Creed F (2007). Maternal depression increases infant risk of diarrhoeal illness: –a cohort study. Arch Dis Child.

[18] Rondó PHC, Ferreira RF, Nogueira F, Ribeiro MCN, Lobert H, Artes R (2003). Maternal psychological stress and distress as predictors of low birth weight, prematurity and intrauterine growth retardation. Eur J Clin Nutr.

[19] Patel V, Prince M (2006). Maternal psychological morbidity and low birth weight in India. Br J Psychiatry.

[20] Fonsah JY, Njamnshi AK, Kouanfack C, Qiu F, Njamnshi DM, Tagny CT, Nchindap E, Kenmogne L, Mbanya D, Heaton R, Kanmogne GD (2017). Adherence to antiretroviral therapy (ART) in Yaoundé-Cameroon: association with opportunistic infections, depression, ART regimen and side effects. PLoS One.

[21] Rueda S, Mitra S, Chen S, Gogolishvili D, Globerman J, Chambers L, Wilson M, Logie CH, Shi Q, Morassaei S, Rourke SB (2016). Examining the associations between HIV-related stigma and health outcomes in people living with HIV/AIDS: a series of meta-analyses. BMJ Open.

[22] Brittain K, Mellins CA, Phillips T, Zerbe A, Abrams EJ, Myer L, Remien RH (2017). Social support, stigma and antenatal depression among HIV-infected pregnant women in South Africa. AIDS Behav.

[23] Fang X, Vincent W, Calabrese SK, Heckman TG, Sikkema KJ, Humphries DL, Hansen NB (2015). Resilience, stress, and life quality in older adults living with HIV/AIDS. Aging Ment Health.

[24] Andini Sandra, Yona Sri, Waluyo Agung (2019). Self-efficacy, depression, and adherence to antiretroviral therapy (ART) among Indonesian women with HIV. Enfermería Clínica.

[25] WHO (2014). Mental health atlas country profile – Tanzania.

[26] Rahman A, Malik A, Sikander S, Roberts C, Creed F (2008). Cognitive behaviour therapy-based intervention by community health workers for mothers with depression and their infants in rural Pakistan: a cluster-randomised controlled trial. Lancet.

[27] Chibanda D, Mesu P, Kajawu L, Cowan F, Araya R, Abas MA (2011). Problem-solving therapy for depression and common mental disorders in Zimbabwe: piloting a task-shifting primary mental health care intervention in a population with a high prevalence of people living with HIV. BMC Public Health.

[28] Petersen I, Bhana A, Baillie K, MhaPP Research Programme Consortium (2012). The feasibility of adapted group-based interpersonal therapy (IPT) for the treatment of depression by community health workers within the context of task shifting in South Africa. Community Ment Health J.

[29] Selohilwe O, Bhana A, Garman EC, Petersen I (2019). Evaluating the role of levels of exposure to a task shared depression counselling intervention led by behavioural health counsellors: outcome and process evaluation. Int J Ment Health Syst.

[30] Rahman A, Khan MN, Hamdani SU, Chiumento A, Akhtar P, Nazir H, Nisar A, Masood A, Din IU, Khan NA, Bryant RA, Dawson KS, Sijbrandij M, Wang D, van Ommeren M (2019). Effectiveness of a brief group psychological intervention for women in a post-conflict setting in Pakistan: a single-blind, cluster, randomised controlled trial. Lancet.

[31] Ministry of Health, Community Development, Gender, Elderly and Children - MoHCDGEC/Tanzania Mainland, Ministry of Health - MoH/Zanzibar, National Bureau of Statistics - NBS/Tanzania, Office of Chief Government Statistician - OCGS/Zanzibar, and ICF (2016). Tanzania demographic and health survey and malaria indicator survey 2015-2016.

[32] Manyahi J, Jullu BS, Abuya MI (2015). Prevalence of HIV and syphilis infections among pregnant women attending antenatal clinics in Tanzania, 2011. BMC Public Health.

[33] Smith Fawzi MC, Ngakongwa F, Liu Y, Rutayuga T, Siril H, Somba M, Kaaya SF (2019). Validating the patient health questionnaire-9 (PHQ-9) for screening of depression in Tanzania. Neurol Psychiatr Brain Res.

[34] Kaaya SF, Blander J, Antelman G (2013). Randomized controlled trial evaluating the effect of an interactive group counseling intervention for HIV-positive women on prenatal depression and disclosure of HIV status. AIDS Care.

[35] Chibanda D, Cowan FM, Healy JL, Abas M, Lund C (2015). Psychological interventions for common mental disorders for people living with HIV in low- and middle-income countries: systematic review. Trop Med Int Health.

[36] Smith Fawzi MC, Siril H, Liu Y, Keith McAdam K, Ainebyona D, McAdam E, Somba M, Oljemark K, Mleli N, Lienert J, Andrew I, Haberlen S, Simwinga A, Todd J, Makongwa S, Li N, Kaaya S (2019). Agents of change: stepped wedge randomized controlled trial of the *NAMWEZA* intervention with people living with HIV and members of their social networks. BMJ Global Health.

[37] Siril HN, Kaaya SF, Smith Fawzi MC, Mtisi E, Somba M, Kilewo J, Mugusi F, Minja A, Kaale A, Todd J (2017). Clinical outcomes and loss to follow-up among people living with HIV participating in the NAMWEZA intervention in Dar es Salaam, Tanzania: a prospective cohort study. AIDS Res Ther.

[38] World Health Organization (2015). Thinking healthy: a manual for psychosocial management of perinatal depression (WHO generic field-trial version 1.0).

[39] World Health Organization (2010). Intervention guide for mental, neurological and substance use disorders in non-specialized health settings: Mental Health Gap Action Programme (mhGAP).

[40] Kroenke K, Spitzer RL, Williams JBW (2001). The PHQ-9: validity of a brief depression severity measure. J Gen Intern Med.

[41] Tanzania Commission for AIDS - TACAIDS, Zanzibar AIDS Commission - ZAC/Tanzania, National Bureau of Statistics - NBS/Tanzania, Office of the Chief Government Statistician - OCGS/Tanzania, and ICF International (2013). Tanzania HIV/AIDS and malaria indicator survey 2011–12.

[42] Broadhead WE, Gehlbach SH, de Gruy FV, Kaplan BH (1988). The Duke-UNC functional social support questionnaire. Measurement of social support in family medicine patients. Med Care.

[43] Schwarzer R, Bassler J, Kwiatek P, Schroder K (1997). The assessment of optimistic self-beliefs: comparison of the German, Spanish, and Chinese versions of the general self-efficacy scale. Appl Psychology.

[44] Siril H, Smith Fawzi MC, Todd J, Wyatt M, Kilewo J, Ware N, Kaaya S (2017). Hopefulness fosters affective and cognitive constructs for actions to cope and enhance quality of life among people living with HIV in Dar Es Salaam, Tanzania. J Int Assoc Provid AIDS Care.

[45] Berger BE, Ferrans CE, Lashley FR (2001). Measuring stigma in people with HIV: psychometric assessment of the HIV stigma scale. J Res Nurs Health.

[46] Surkan PJ, Mukherjee JS, Williams DR (2010). Perceived discrimination and stigma toward children affected by HIV/AIDS and their HIV-positive caregivers in central Haiti. AIDS Care.

[47] Ivers LC, Teng JE, Gregory Jerome J, Bonds M, Freedberg KA, Franke MF (2014). A randomized trial of ready-to-use supplementary food versus corn-soy blend plus as food rations for HIV-infected adults on antiretroviral therapy in rural Haiti. Clin Infect Dis.

[48] Safren Steven A., Biello Katie B., Smeaton Laura, Mimiaga Matthew J., Walawander Ann, Lama Javier R., Rana Aadia, Nyirenda Mulinda, Kayoyo Virginia M., Samaneka Wadzanai, Joglekar Anjali, Celentano David, Martinez Ana, Remmert Jocelyn E., Nair Aspara, Lalloo Umesh G., Kumarasamy Nagalingeswaran, Hakim James, Campbell Thomas B. (2014). Psychosocial Predictors of Non-Adherence and Treatment Failure in a Large Scale Multi-National Trial of Antiretroviral Therapy for HIV: Data from the ACTG A5175/PEARLS Trial. PLoS ONE.

[49] Stata Corporation. Stata, version 14.2. College Station. https://www.stata.com/stata14/. Accessed 30 Oct 2019.

[50] Hayes RJ, Moulton LH. Cluster Randomised Trials. CRC Press. https://www.crcpress.com/Cluster-Randomised-Trials/Hayes-Moulton/p/book/9781584888178. Published January 12, 2009. Accessed 11 June 2018.

[51] Sin NL, DiMatteo MR (2014). Depression treatment enhances adherence to antiretroviral therapy: a meta-analysis. Ann Behav Med.

[52] Arseniou S, Arvaniti A, Samakouri M (2014). HIV infection and depression. Psychiatry Clin Neurosci.

[53] Raposa EB, Hammen CL, Brennan PA, O’Callaghan F, Najman JM (2014). Early adversity and health outcomes in young adulthood: The role of ongoing stress. Health Psychol.

[54] Rael CT, Davis A (2017). Elevated depression symptoms and key associated factors in female sex workers and women living with HIV/AIDS in the Dominican Republic. Int J STD AIDS.

[55] Yeneabat T, Bedaso A, Amare T (2017). Factors associated with depressive symptoms in people living with HIV attending antiretroviral clinic at Fitche Zonal Hospital, Central Ethiopia: cross-sectional study conducted in 2012. Neuropsychiatr Dis Treat.

[56] Tsai AC, Bangsberg DR, Frongillo EA, Hunt PW, Muzoora C, Martin JN, Weiser SD (2012). Food insecurity, depression and the modifying role of social support among people living with HIV/AIDS in rural Uganda. Soc Sci Med.

[57] Wong VJ, Murray KR, Phelps BR, Vermund SH, McCarraher DR (2017). Adolescents, young people, and the 90–90–90 goals: a call to improve HIV testing and linkage to treatment. AIDS.

[58] Vyavaharkar M, Moneyham L, Corwin S, Saunders R, Annang L, Tavakoli A (2010). Relationships between stigma, social support, and depression in HIV-infected African American women living in the rural Southeastern United States. J Assoc Nurses AIDS Care.

[59] Grote NK, Zuckoff A, Swartz H, Bledsoe SE, Geibel S (2007). Engaging women who are depressed and economically disadvantaged in mental health treatment. Soc Work.

[60] Lloyd M, Ramon S, Vakalopoulou A (2017). Women’s experiences of domestic violence and mental health: findings from a European empowerment project. Psychol Violence.

[61] Weissman MM, Wickramaratne P, Pilowsky DJ (2015). Treatment of maternal depression in a medication clinical trial and its effect on children. Am J Psychiatry.

